# Diagnostic value of contrast-enhanced ultrasound in hepatocellular carcinoma: a meta-analysis with evidence from 1998 to 2016

**DOI:** 10.18632/oncotarget.20049

**Published:** 2017-08-07

**Authors:** Juanjuan Zhang, Yanyan Yu, Ying Li, Lunshou Wei

**Affiliations:** ^1^ Department of Ultrasound, Huaihe Hospital of Henan University, Kaifeng, China; ^2^ Department of Gastroenterology, Huaihe Hospital of Henan University, Kaifeng, China

**Keywords:** hepatocellular carcinoma, contrast-enhanced ultrasound, diagnostic value, meta-analysis

## Abstract

**Background:**

This meta-analysis is aimed at determining the diagnostic value of hepatocellular carcinoma (HCC) with contrast-enhanced ultrasound (CEUS).

**Materials and Methods:**

A comprehensive literature search of Pubmed, Web of Science, and the Cochrane Library was performed to identify published studies. The methodological quality of the included studies was evaluated. Data from eligible studies were used to estimate the pooled sensitivity, specificity, diagnostic odds ratio (DOR), positive and negative likelihood ratio (LR) and summary receiver operating characteristic (SROC) curve. Meta-Disc and STATA softwares were utilized for all statistical analyses.

**Results:**

Fifty-three eligible studies (publication years ranged from 1998 to 2016) were selected according to inclusion criteria. The meta-analysis showed that the pooled sensitivity and specificity of CEUS to detect HCC were 0.85 (95% CI: 0.84–0.86) and 0.91 (95% CI: 0.90–0.92), respectively. The pooled positive and negative LRs were 6.28 (95% CI: 4.49–8.77) and 0.16 (95% CI: 0.12–0.22), respectively. The pooled DOR was 55.01 (95% CI: 35.25–83.47). The area under the SCOR curve was 0.9432. Meta-regression and funnel plot indicated that sample size, type of contrast agents and publication bias might be the major sources of heterogeneity.

**Conclusions:**

CEUS is a valuable diagnostic tool for identifying HCC in clinic with highly sensitive and specific.

## INTRODUCTION

According to the statistics, hepatocellular carcinoma (HCC) ranks the fifth most common cancer in men, the seventh in women [1]. An estimated 782,500 new liver cancer cases and 745,500 deaths occurred worldwide during 2012, with China alone accounting for about 50% of the total number of cases and deaths [2]. Nowadays, HCC remains the life-threatening complaint despite the advanced surgical procedures and other nonoperative methods, with 5-year overall survival rate is less than 10% in its advanced stage [3]. Hence, early accurate diagnosis should be performed to improve patient’s prognosis. Thanks to the typical hemodynamic changes of HCC (hypervascularity in the arterial phase followed by “washout” on portal or delayed phases), [4] the noninvasive diagnosis through imaging examinations could be reached without histologic confirmation.

However, currently established guidelines by the Association for the Study of Liver Diseases (AASLD), Liver Imaging Reporting and Data System (LI-RADS), and the Asian-Pacific Association for the Study of the Liver (APASL) all endorse multi-detector spiral CT and MRI with contrast agents as the first line modalities for diagnosing HCC [5–7], although ultrasound (US) is the more often used modality to monitoring the patients with hepatic cirrhosis who are at high risk for HCC in clinical practice [4]. In recent years, micro-bubble based contrast agents have greatly improved the sensitivity as well as specificity for characterization of focal liver lesions (FLLs) during the ultrasound examinations [8]. While, the role of contrast-enhanced ultrasound (CEUS) as a first line procedure for diagnosis of HCC still remains controversial. Some physicians are not convinced regarding the value of CEUS for the diagnosis of HCC. To this end, this systematic review and meta-analysis is conducted to evaluate the diagnostic value of CEUS in HCC.

## RESULTS

### Identification of eligible studies

**Figure 1:**
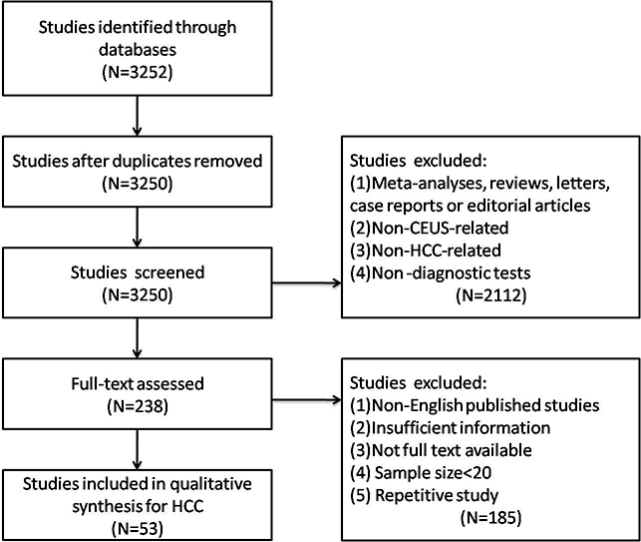
Flow chart of study selection

A comprehensive literature search was conducted and revealed 3252 primary studies. Two studies were excluded due to duplicated publications. After reviewing the titles and abstracts, 2112 studies were excluded with the reasons as follows: (1) non-prospective / retrospective articles (meta-analyses, reviews, letters, case reports or editorial articles); (2) non-CEUS-related articles; (3) non-HCC related articles; (4) non-diagnostic tests. The remaining 238 articles were further assessed by screening the full texts, among which 185 articles were excluded due to (1) non-English published studies; (2) insufficient information; (3) not full text available; (4) sample size < 20; (5) repetitive study. Eventually, 53 eligible articles [9–61] were included in this meta-analysis. Detailed selection process was illustrated in a flow chart (Figure 1).

### Study characteristics and quality assessment

The detailed characteristics of the 53 studies are presented in Supplementary Table 1. The average age of the patients included in the eligible studies ranged from 44 to 71. And there were 5977 lesions in total, with more than 4827 patients (the numbers of patients were not mentioned in two studies). The publication years ranged from 1998 to 2016. There were 34 studies conducted in Asian population, and 19 studies conducted in European and American population. The second generation contrast agents for CEUS (SonoVue, Sonazoid and Definity) were used in 40 studies along with the first generation contrast agent (Levovist) in 13 studies. The methodological quality of the included 53 studies is also summarized in Supplementary Table 1.

### Diagnostic accuracy of HCC

**Figure 2:**
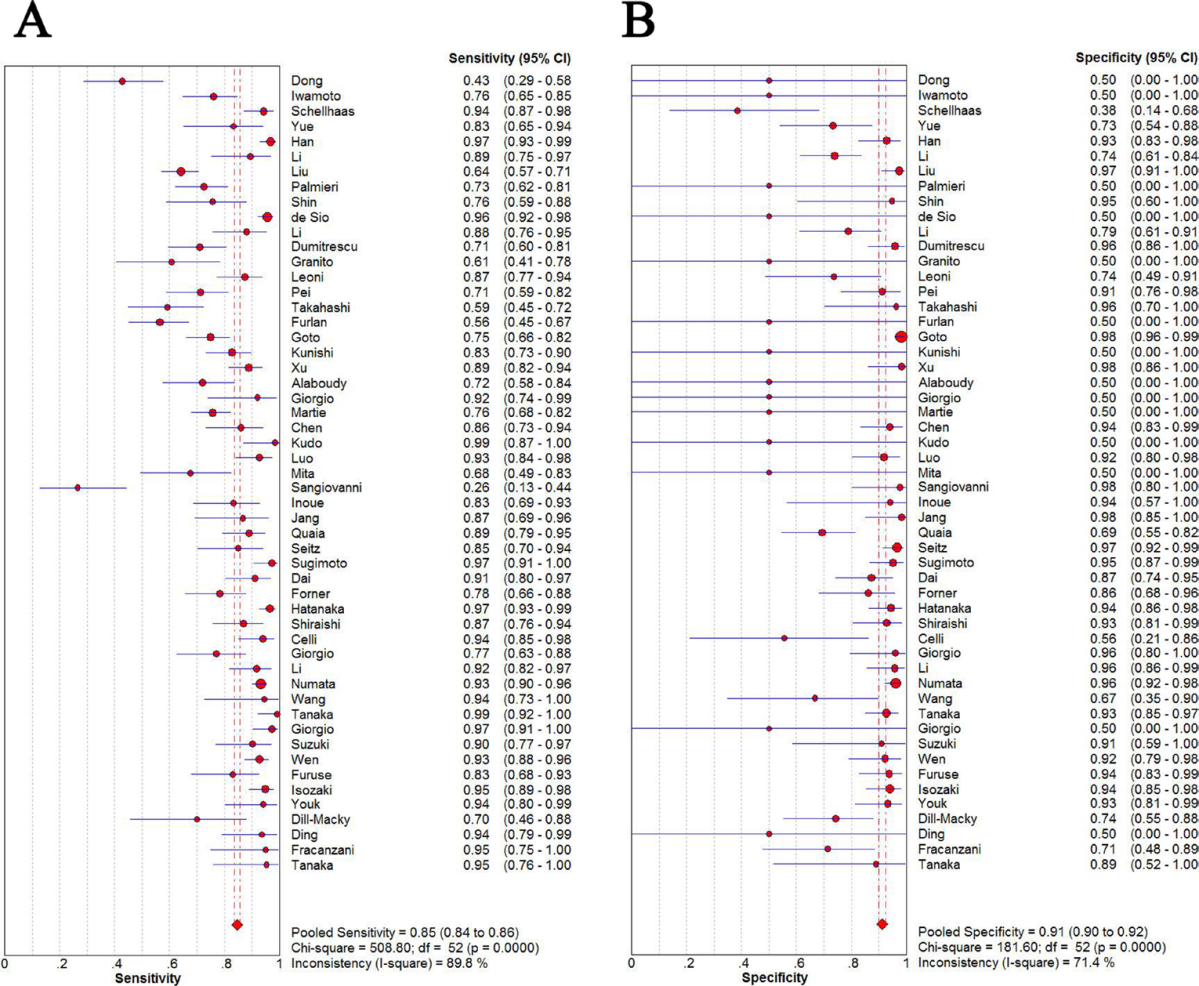
Sensitivity (A) and specificity (B) of diagnosis of HCC with CEUS

**Figure 3:**
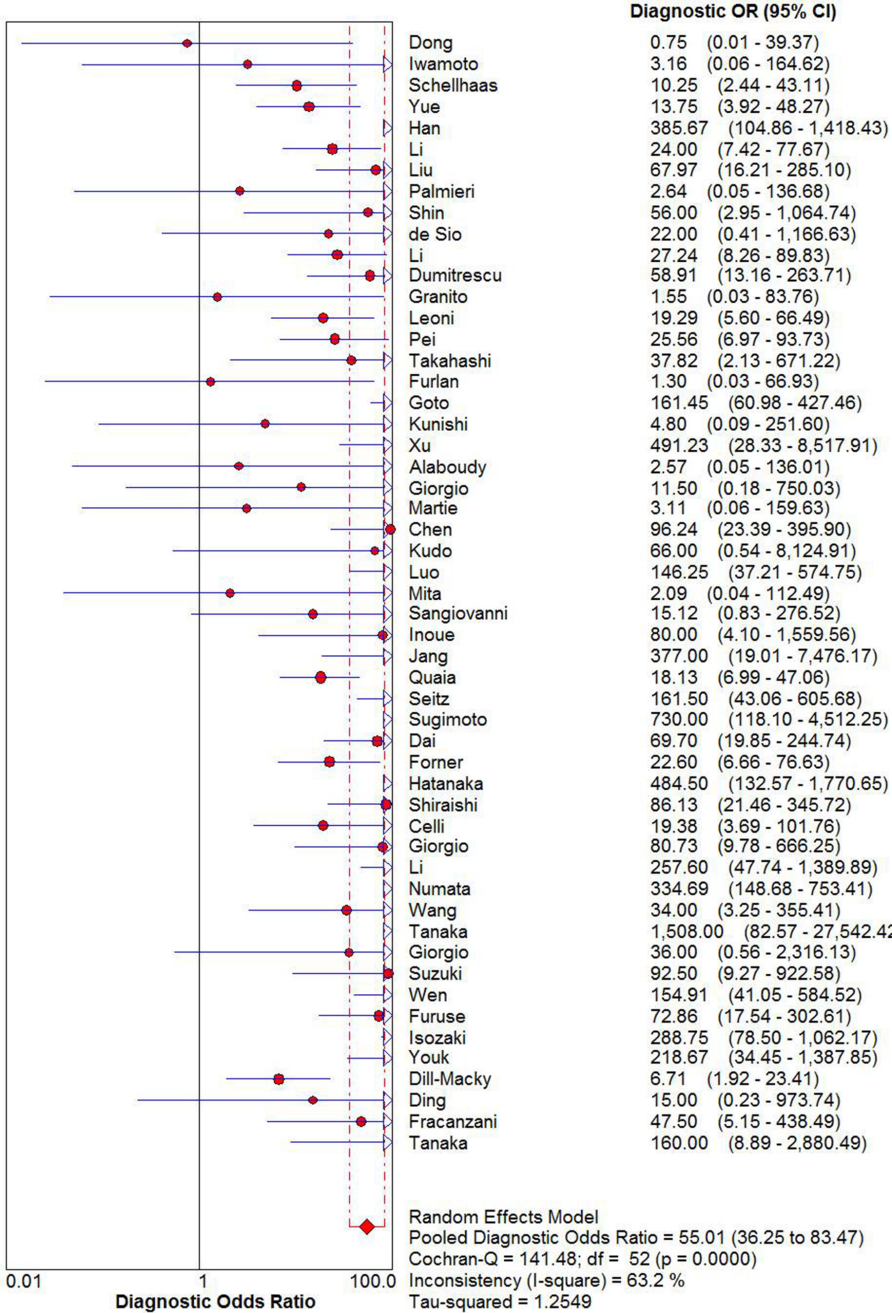
DOR of diagnosis of HCC with CEUS

**Figure 4:**
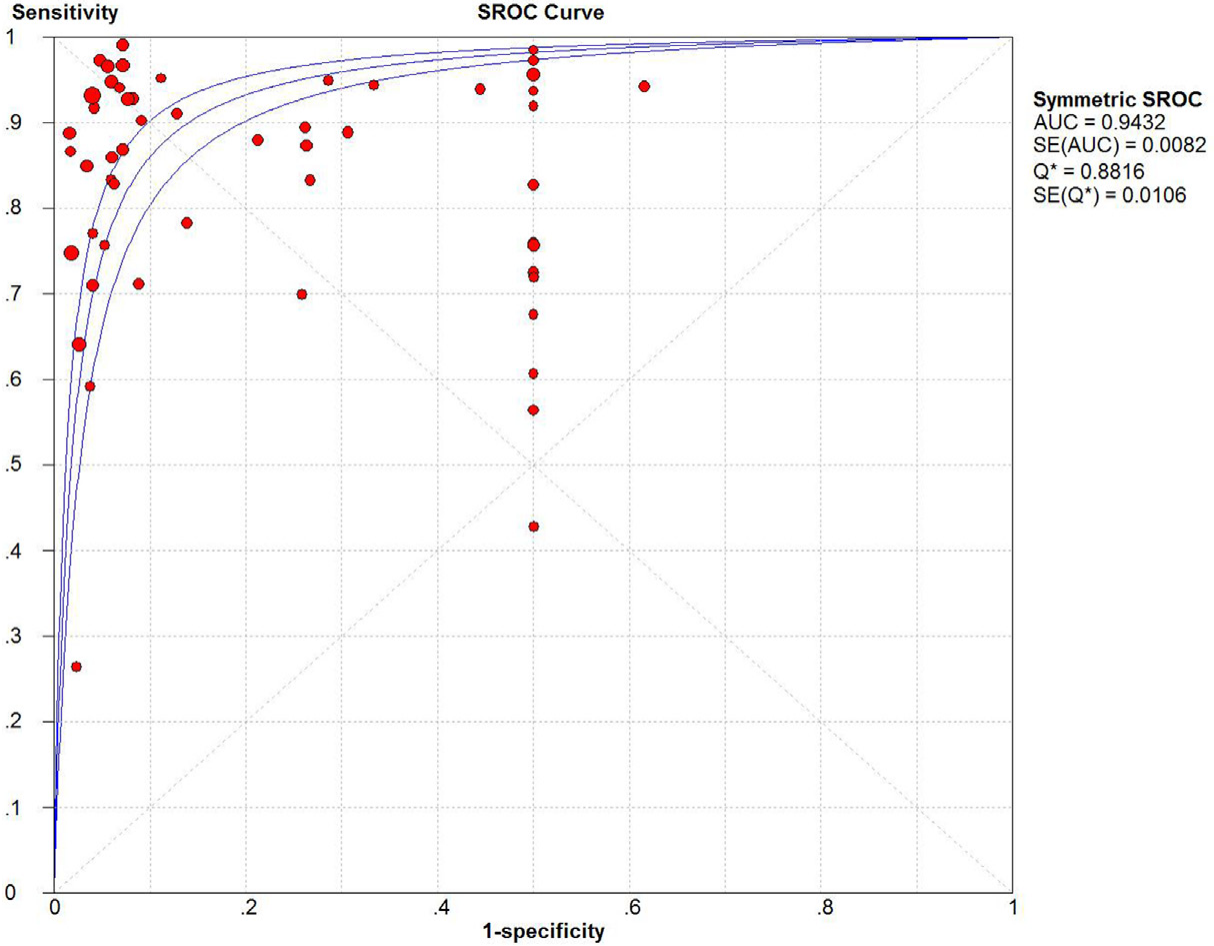
SROC curve of CEUS

The pooled sensitivity and specificity of CEUS for the diagnosis of HCC were 0.85 (95% CI: 0.84 - 0.86) and 0.91 (95% CI: 0.90 - 0.92), respectively (Figure 2). The pooled positive and negative LRs were 6.28 (95% CI: 4.49 - 8.77) and 0.16 (95% CI: 0.12 - 0.22), respectively. The pooled DOR was 55.01 (95% CI: 35.25 - 83.47) (Figure 3). The meta-analysis results showed out that CEUS had high discriminatory powers of positive and negative test results. The SROC curve was illustrated in Figure 4. AUC of CEUS and the Q* index were 0.9432 and 0.8816, respectively, which were close to 1. Thus, they indicated CEUS was a useful diagnostic tool to distinguish HCC from other liver lesions.

### Heterogeneity results

As seen in the forest plots (Figure 2 and Figure 3), all indices of diagnostic accuracy denoted heterogeneity. Spearman correlation coefficient showed there was no significant correlation between sensitivity and specificity (*r* = 0.060, *P* = 0.669), which indicated no threshold effect. To further explore the sources of heterogeneity, meta-regression analysis was performed based on district (group1: Europe and America, group2: Asia), sample size (group 1: *n* < 100, group 2: *n* ≥ 100) and type of contrast agents (group 1: SonoVue, group 2: Sonazoid, group 3: Definity, group 4: Levovist). The results indicated that sample size and type of contrast agents might be the major sources of heterogeneity (*P* = 0.002, *P* = 0.009, respectively) (Table 1).

**Table 1:** Meta-regression analysis of potential source of heterogeneity

Potential sources	*P* value	RDOR	UL	LL
District	0.2515	1.56	0.72	3.37
Sample size	0.002	4.24	2.09	8.61
Contrast agent	0.009	1.93	1.33	2.80

RDOR relative diagnostic odds ratio, UL upper limit, LL lower limit.

**Figure 5:**
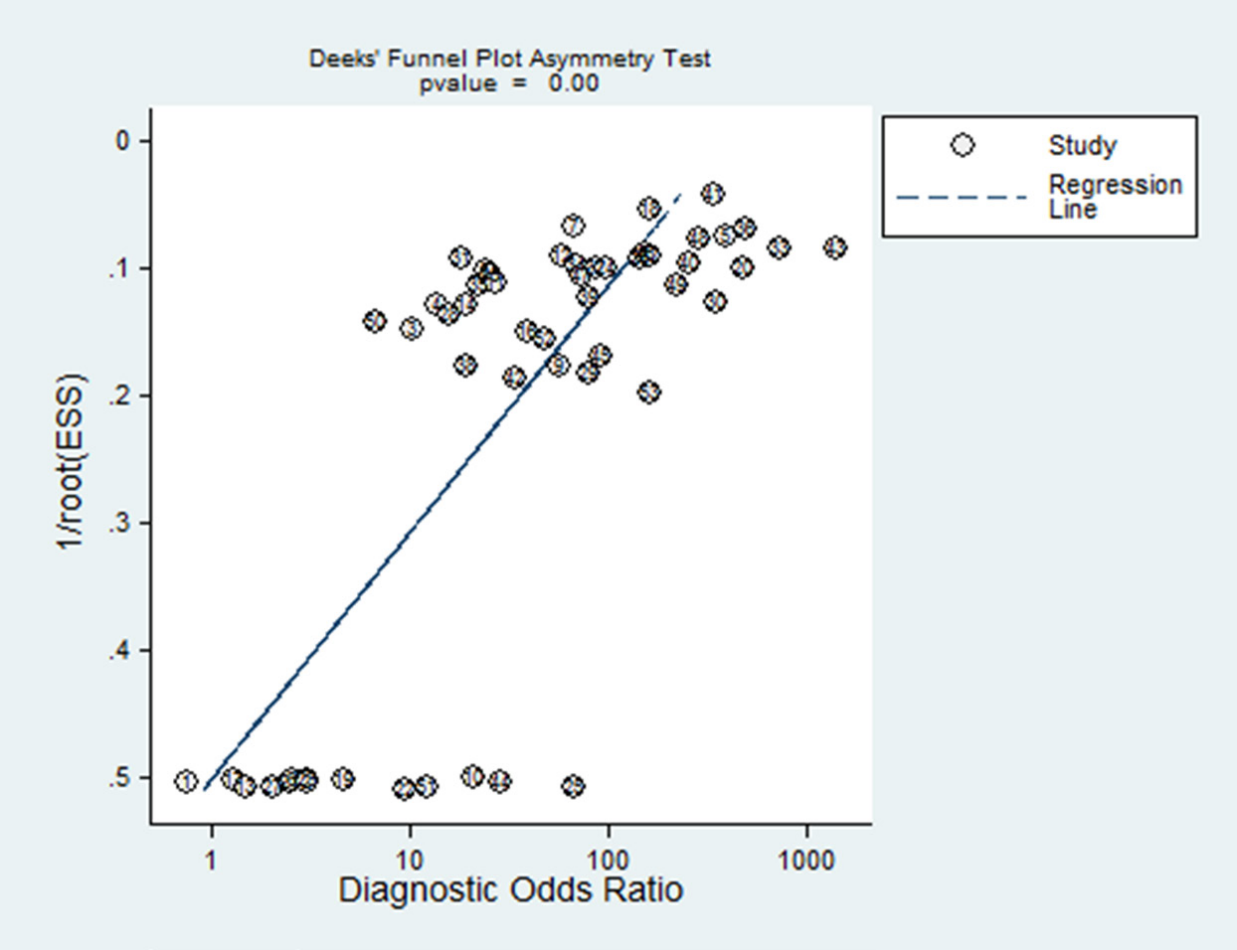
Funnel plot of publication bias on the pooled DOR

Funnel plot was conducted to assess the publication bias of the eligible studies. However, as seen in Figure 5, the plot was asymmetric indicating that the publication bias existed (*P* = 0.000). This indicated publication bias might be another source of heterogeneity.

## DISCUSSION

The utilization of microbubble-based ultrasound contrast agents along with the advanced US imaging techniques now allows stable observation and detailed evaluation of the tissue macro and microvascularization in both qualitative and quantitative manner [62]. According to other literatures, the ability of CEUS is similar to contrast-enhanced computed tomography (CECT) and contrast-enhanced resonance magnetic imaging (CEMRI) [63–64]. Beyond that, CEUS has unique advantages over CECT and CEMRI in the characterization of hepatic lesions. These include the capability of real-time dynamic imaging, repetitive observation of tumor vascularity with multiple injections of these contrast agents, and the unique intravascular properties with excellent safety profile of the microbubbles, which allow the applications in patients with decreased renal function [8]. Although, the Food and Drug Administration (FDA) in U.S. finally approved their applications for a noncardia use in 2016, CEUS has been widely used for liver imaging especially for characterizing the HCCs in European and Asian countries for more than ten years [65]. This license might result in a possible breakthrough in the field of CEUS study.

The HCC multistep carcinogenesis (from regenerative nodule to dysplastic nodule, ending with HCC) leads to changes in blood supply within the nodule, which eventually forms increased tumoral arterial supply along with decreased normal arterial supply and portal supply as a consequent [66]. Thus, HCC is characterized by arterial phase hypervascularity followed by later and low washout on CEUS. Several researchers suggest that CEUS is superior to CT or MRI due to the real-time observation of arterial phase enhancement which might be missed by CT or MRI because of the predetermined scanning delay [67–68]. In our meta-analysis which performed on 53 eligible studies, the pooled sensitivity, specificity and DOR of CEUS for diagnosis of HCC were 0.85, 0.91 and 55.01, respectively. The pooled positive and negative LRs were 6.28 and 0.16, respectively. All the results implied that CEUS might be the excellent choice in the diagnostic work-up of liver malignant lesions. Nevertheless, there still exist some disadvantages of CEUS. The main drawback is the operator-dependency in US examination. While our comprehensive meta-analysis showed good pooled diagnostic values with relatively narrow confidence intervals, indicating with certain skills and experience, most of the sonographers might achieve closely high diagnostic capability.

In our meta-analysis, great heterogeneity was revealed. While there was no explicit threshold effect in this meta-analysis, indicating that the threshold effect was not the source of heterogeneity. Therefore, meta-regression analysis was performed to further explore the sources. And the results showed sample size and type of contrast agents might be the major sources of heterogeneity. Publication bias was also detected in this meta-analysis, suggesting it might be another source of heterogeneity.

Moreover, some limitations in our meta-analysis should be acknowledged. Firstly, due to the publication bias explored in this meta-analysis, the pooled estimates might be more optimistic than they actually are, as studies with positive data are more likely to be published. Secondly, some studies indicated that the vascularity in small nodule could not be easily assessed by CEUS [69]. But since the data on small nodules couldn’t be obtained in most of the eligible studies, the diagnostic value of CEUS for small HCC could not be estimated at present.

In conclusion, this comprehensive meta-analysis demonstrates the magnitude of the importance of CEUS in the diagnosis of HCC. Because of the advantages mentioned above, this approach would offer a major role in the diagnosis area, and additionally CEUS might become a first-line imaging tool in the future.

## MATERIALS AND METHODS

### Literature search strategies

A comprehensive literature search of studies was carried out to identify eligible articles from the electronic databases, including Pubmed, Web of Science, and the Cochrane Library, up to February 1st, 2017, and no limit to the starting time. The search terms included “hepatocellular carcinoma” OR “hepatic tumor” OR “liver tumor” OR “hepatic cancer” OR “liver cancer”, AND “contrast-enhanced ultrasound” OR “contrast-enhanced ultrasonography” OR “contrast-enhanced US” OR “CEUS” OR “contrast-enhanced Doppler ultrasonography”. Additional relevant search was also performed by manually searching the references of eligible studies and relevant reviews.

### Study selection

Two reviewers separately selected the eligible studies with disagreements disposed by consensus. Studies were considered eligible if they fulfilled the inclusion criteria: (1) full article published in English with the full text available; (2) articles dealing with the diagnosis of contrast-enhanced ultrasound for HCC; (3) articles confirmed the diagnosis with the reference standard as histopathologic analysis and/or close clinical diagnosis with imaging follow-up; (4) published data in the fourfold (2 × 2) tables or articles presented sufficient data to calculate the true-positive (TP), true-negative (TN), false-positive (FP) and false-negative (FN); (5) at least 20 patients were included in the study. Studies were excluded when they were (1) meta-analyses, reviews, letters, case reports or editorial articles; (2) not clinical studies; (3) not using CEUS to diagnose HCC; (4) the patients were not confirmed the diagnosis with the above standards. If there existed more than one study by the same authors using the same cases published, either the most recently published studies or the study with the largest sample size was included.

### Data extraction

All eligible studies were used for data extraction by two reviewers independently. Disagreements were disposed by a third reviewer. The following characteristics were extracted from the eligible studies: first author, publication year, country, number of lesions and patients, clinical characteristics of the study sample (age and gender ratio), gold standard, contrast agent, TP, TN, FP and FN.

### Quality assessment

The methodological quality of eligible studies was assessed by the quality assessment tool for diagnostic accuracy studies (QUADAS). It contains fourteen assessment items, each of which was assessed as “yes” (1 score) or “no” (0 score) or “unclear” (-1 score).

### Statistical analysis

Softwares of Meta-Disc (version 1.4, Universidad Complutense, Madrid, Spain) and STATA (version 11.0, STATA Corporation, College Station, Texas, USA) were used for all statistical analyses. For each study, we constructed 2 × 2 contingency tables wherein all subjects were classified to have positive or negative CEUS results. If one contingency table had a cell with no events, we added 0.5 to all cells. We calculated the sensitivity as TP/(TP+FN), the specificity as TN/(TN+FP), the positive likelihood ratio (PLR) as sensitivity/(1-specificity), the negative likelihood ratio (NLR) as (1-sensitivity)/specificity, and the diagnostic odds ratio (DOR) as (TP × TN)/(FP × FN) along with their 95% confidence intervals (CIs). The summary receiver operating characteristic (SROC) curve, the area under the curve (AUC) as well as the Q* index were calculated. The threshold effect was tested by the Spearman correlation coefficient. The heterogeneity was assessed by Cochran’s *Q* statistic and *I*^2^ test. When *I*^2^ ≥ 50% or *P*_*heterogeneity*_ < 0.05, the random effect model was used in this meta-analysis, otherwise the fixed effect model was used. Potential sources of heterogeneity were explored by regression analysis. The potential publication bias was assessed by the funnel plot. *P* values were two-sided and difference was considered as statistically significant when *P* < 0.05.

## SUPPLEMENTARY MATERIALS TABLE





## Abbreviations

HCC: Hepatocellular carcinoma
CEUS: contrast-enhanced ultrasound
AASLD: Association for the Study of Liver Diseases
LI-RADS: Liver Imaging Reporting and Data System
APASL: Asian-Pacific Association for the Study of the Liver
US: ultrasound
FLLs: focal liver lesions
TP: true-positive
TN: true-negative
FP: false-positive
FN: false-negative
QUADAS: quality assessment tool for diagnostic accuracy studies
PLR: positive likelihood ratio
NLR: negative likelihood ratio
DOR: diagnostic odds ratio
CIs: confidence intervals
SROC: summary receiver operating characteristic
AUC: area under the curve
