# Supplementary material for: Diagnostic value of contrast-enhanced ultrasound in hepatocellular carcinoma: a meta-analysis with evidence from 1998 to 2016
Source: Oncotarget. 2017 Aug 7;8(43):75418–26. doi: 10.18632/oncotarget.20049 (PMC5650432; doi:10.18632/oncotarget.20049)
Supplement: Supplementary file 1 [file oncotarget-08-75418-s001.pdf]

# Diagnostic value of contrast-enhanced ultrasound in hepatocellular carcinoma: a meta-analysis with evidence from 1998 to 2016

## SUPPLEMENTARY MATERIALS

**Supplementary Table 1: The characteristics and QUADAS score of eligible studies.** See Supplementary_Table_1
