# Supplementary material for: Diagnostic value of contrast-enhanced ultrasound in hepatocellular carcinoma: a meta-analysis with evidence from 1998 to 2016
Source: Oncotarget. 2017 Aug 7;8(43):75418–26. doi: 10.18632/oncotarget.20049 (PMC5650432; doi:10.18632/oncotarget.20049)
Supplement: Supplementary file 2 [file oncotarget-08-75418-s002.docx]

**Supplementary Table 1: The characteristics and QUADAS score of eligible studies.**

| Author | Year | Country | Number of lesions / number of patients | Gender  (male/female) | Average age | Contrast agent | TP | FP | FN | TN | QUADAS score |
| --- | --- | --- | --- | --- | --- | --- | --- | --- | --- | --- | --- |
| Dong^[9]^ | 2016 | China | 49 / 41 | 37 / 4 | 53 | SonoVue | 21 | 0 | 28 | 0 | 9 |
| Iwamoto^[10]^ | 2016 | Japan | 79 / 77 | 56 / 21 | 70 | Sonazoid | 60 | 0 | 19 | 0 | 8 |
| Schellhaas^[11]^ | 2016 | Germany | 100 /100 | 85 / 15 | 66 | SonoVue | 82 | 8 | 5 | 5 | 10 |
| Yue^[12]^ | 2016 | China | 60 / 60 | 39 / 21 | NA | SonoVue | 25 | 8 | 5 | 22 | 9 |
| Han^[13]^ | 2015 | China | 240 / 240 | 196 / 44 | NA | Sonovue | 178 | 4 | 6 | 52 | 9 |
| Li^[14]^ | 2015 | China | 103 / 103 | 72 / 31 | 44 | SonoVue | 34 | 17 | 4 | 48 | 9 |
| Liu^[15]^ | 2015 | China | 276/ NA | NA | NA | SonoVue | 127 | 2 | 71 | 76 | 11 |
| Palmieri^[16]^ | 2015 | Italy | 90 /85 | NA | NA | SonoVue | 66 | 0 | 25 | 0 | 10 |
| Shin^[17]^ | 2015 | Korea | 46 / 46 | 34 / 12 | 58 | SonoVue | 28 | 0 | 9 | 9 | 9 |
| de Sio^[18]^ | 2014 | Italy | 230 / NA | NA | NA | SonoVue | 220 | 0 | 10 | 0 | 10 |
| Li^[19]^ | 2014 | China | 83 / 83 | 72 / 11 | NA | SonoVue | 44 | 7 | 6 | 26 | 12 |
| Dumitrescu^[20]^ | 2013 | Romania | 126 / 126 | 75 / 51 | 59 | SonoVue | 54 | 2 | 22 | 48 | 10 |
| Granito^[21]^ | 2013 | Italy | 38 / 28 | NA | NA | SonoVue | 17 | 0 | 11 | 0 | 10 |
| Leoni^[22]^ | 2013 | Italy | 90 / 81 | NA | NA | SonoVue | 62 | 5 | 9 | 14 | 12 |
| Pei^[23]^ | 2013 | China | 100 / 100 | 41 / 29 | NA | SonoVue | 47 | 3 | 19 | 31 | 11 |
| Takahashi^[24]^ | 2013 | Japan | 67 / 56 | 40 / 16 | 66 | Sonazoid | 32 | 0 | 22 | 13 | 7 |
| Furlan^[25]^ | 2012 | Italy | 85 / 82 | NA | NA | SonoVue | 48 | 0 | 37 | 0 | 8 |
| Goto^[26]^ | 2012 | Japan | 400 /100 | 60 / 40 | 68 | Sonazoid | 92 | 5 | 31 | 272 | 8 |
| Kunishi^[27]^ | 2012 | Japan | 87 / 50 | 35 / 15 | 71 | Sonazoid | 72 | 0 | 15 | 0 | 7 |
| Xu^[28]^ | 2012 | China | 147 / 133 | 111 / 22 | 52 | SonoVue | 103 | 0 | 13 | 31 | 9 |
| Alaboudy^[29]^ | 2011 | Japan | 50 / 32 | 23 / 9 | 68 | Sonazoid | 36 | 0 | 14 | 0 | 10 |
| Giorgio^[30]^ | 2011 | Italy | 40 / 40 | NA | 60 | SonoVue | 23 | 0 | 2 | 0 | 9 |
| Martie^[31]^ | 2011 | Romania | 148 / 100 | 69 / 31 | NA | SonoVue | 112 | 0 | 36 | 0 | 9 |
| Chen^[32]^ | 2010 | China | 100 / 100 | 77 / 23 | NA | SonoVue | 43 | 3 | 7 | 47 | 11 |
| Kudo^[33]^ | 2010 | Japan | 33 / 33 | NA | NA | Sonazoid | 33 | 0 | 0 | 0 | 12 |
| Luo^[34]^ | 2010 | Japan | 119 / 119 | 67 / 52 | NA | Sonazoid | 65 | 4 | 5 | 45 | 9 |
| Mita^[35]^ | 2010 | Japan | 34 / 29 | 13 / 16 | 71 | Sonazoid | 23 | 0 | 11 | 0 | 10 |
| Sangiovanni^[36]^ | 2010 | Italy | 55 / 34 | NA | NA | SonoVue | 9 | 0 | 25 | 21 | 10 |
| Inoue^[37]^ | 2009 | Japan | 50 / 50 | 38 / 12 | 67 | Levovist | 35 | 0 | 7 | 8 | 10 |
| Jang^[38]^ | 2009 | Canada | 59 / 59 | 43 / 16 | 56 | Definity | 26 | 0 | 4 | 29 | 10 |
| Quaia^[39]^ | 2009 | Italy | 121 / 106 | 68 / 38 | 70 | SonoVue | 64 | 15 | 8 | 34 | 9 |
| Seitz^[40]^ | 2009 | Germany | 154 / 154 | NA | NA | SonoVue | 34 | 4 | 6 | 114 | 7 |
| Sugimoto^[41]^ | 2009 | Japan | 137 / 137 | NA | NA | Sonazoid | 73 | 3 | 2 | 60 | 8 |
| Dai^[42]^ | 2008 | China | 103 / 72 | 59 / 13 | 59 | SonoVue | 51 | 6 | 5 | 41 | 8 |
| Forner^[43]^ | 2008 | Spain | 89 / 89 | 53 / 36 | 65 | SonoVue | 47 | 4 | 13 | 25 | 9 |
| Hatanaka^[44]^ | 2008 | Japan | 249 / 214 | 134 / 80 | 68 | Sonazoid | 171 | 4 | 6 | 68 | 7 |
| Shiraishi^[45]^ | 2008 | Japan | 103 / 97 | NA | NA | SonoVue | 53 | 3 | 8 | 39 | 7 |
| Celli^[46]^ | 2007 | Italy | 75 / 67 | NA | NA | SonoVue | 62 | 4 | 4 | 5 | 10 |
| Giorgio^[47]^ | 2007 | Italy | 73 / 73 | 49 / 24 | 63 | SonoVue | 37 | 1 | 11 | 24 | 12 |
| Li^[48]^ | 2007 | China | 109 / 109 | 72 / 37 | 46 | SonoVue | 56 | 2 | 5 | 46 | 11 |
| Numata^[49]^ | 2006 | Japan | 586 / 586 | 368 / 218 | NA | Levovist | 357 | 8 | 26 | 195 | 12 |
| Wang^[50]^ | 2006 | China | 30 / 30 | 20 / 10 | 55 | Levovist | 17 | 4 | 1 | 8 | 10 |
| Tanaka^[51]^ | 2005 | Japan | 142 / 142 | 71 / 71 | 61 | Levovist | 58 | 6 | 0 | 78 | 9 |
| Giorgio^[52]^ | 2004 | Italy | 74 / 74 | 60 / 14 | 67 | SonoVue | 72 | 0 | 2 | 0 | 7 |
| Suzuki^[53]^ | 2004 | Japan | 52 / 46 | 31 / 15 | 66 | Levovist | 37 | 1 | 4 | 10 | 9 |
| Wen^[54]^ | 2004 | China | 192 / 163 | 104 / 39 | 63 | Levovist | 142 | 3 | 11 | 36 | 9 |
| Furuse^[55]^ | 2003 | Japan | 89 / 82 | 53 / 29 | 61 | Levovist | 34 | 3 | 7 | 45 | 8 |
| Isozaki^[56]^ | 2003 | Japan | 183 / 183 | 121 / 62 | NA | Levovist | 110 | 4 | 6 | 63 | 7 |
| Youk^[57]^ | 2003 | Korea | 78 / 76 | 57 / 19 | 58 | Levovist | 32 | 3 | 2 | 41 | 9 |
| Dill-Macky^[58]^ | 2002 | Canada | 51 / 43 | NA | NA | Levovist | 14 | 8 | 6 | 23 | 9 |
| Ding^[59]^ | 2001 | Japan | 32 / 26 | 24 / 2 | 66 | Levovist | 30 | 0 | 2 | 0 | 10 |
| Fracanzani^[60]^ | 2001 | Italy | 41 / 41 | 30 / 11 | 62 | Levovist | 19 | 6 | 1 | 15 | 8 |
| Tanaka^[61]^ | 1998 | Japan | 30 / 30 | 26 / 4 | 64 | Levovist | 20 | 1 | 1 | 8 | 10 |

TP true-positive, FP false-positive, FN false-negative, TN true-negative, QUADAS quality assessment tool for diagnostic accuracy studies QUADAS, NA not available.
